# Association Between Mental Health and Reproductive System Disorders in Women: A Systematic Review and Meta-analysis

**DOI:** 10.1001/jamanetworkopen.2023.8685

**Published:** 2023-04-18

**Authors:** Nina Zaks, Anita Batuure, Emma Lin, Anna-Sophie Rommel, Abraham Reichenberg, Dorothy Grice, Veerle Bergink, Nathan S. Fox, Behrang Mahjani, Magdalena Janecka

**Affiliations:** 1 Department of Psychiatry, Icahn School of Medicine at Mount Sinai, New York, New York; 2 Rutgers University, Graduate School of Applied and Professional Psychology, Piscataway, New Jersey; 3 Cornell University, Undergraduate Studies, Ithaca, New York; 4 Seaver Autism Center for Research and Treatment, Icahn School of Medicine at Mount Sinai, New York, New York; 5 Department of Environmental Medicine and Public Health, Icahn School of Medicine at Mount Sinai, New York, New York; 6 Division of Tics, OCD and Related Disorders, Icahn School of Medicine at Mount Sinai, New York, New York; 7 The Mindich Child Health and Development Institute, Icahn School of Medicine at Mount Sinai, New York, New York; 8 Friedman Brain Institute, Icahn School of Medicine at Mount Sinai, New York, New York; 9 Department of Psychiatry, Erasmus Medical Centre Rotterdam, Rotterdam, the Netherlands; 10 Department of Obstetrics, Gynecology, and Reproductive Science, Icahn School of Medicine at Mount Sinai, New York, New York; 11 Department of Medical Epidemiology and Biostatistics, Karolinska Institutet, Stockholm, Sweden; 12 Department of Genetic and Genomic Sciences, Icahn School of Medicine at Mount Sinai, New York, New York

## Abstract

**Importance:**

Reproductive system and mental health disorders are commonly comorbid in women. Although the causes of this overlap remain elusive, evidence suggests potential shared environmental and genetic factors associated with risk.

**Objective:**

To investigate the comorbidity between psychiatric and reproductive system disorders, both as broad diagnostic categories and among specific pairs of diagnoses.

**Data Source:**

PubMed.

**Study Selection:**

Observational studies published between January 1980 and December 2019 assessing prevalence of psychiatric disorders in women with reproductive system disorders and prevalence of reproductive system disorders in women with psychiatric disorders were included. The study did not include psychiatric and reproductive disorders triggered by life events (eg, trauma, infection, surgery) to address potential confounding.

**Data Extraction and Synthesis:**

A search yielded 1197 records, of which 50 met the inclusion criteria for the qualitative and 31 for the quantitative synthesis in our study. A random-effects model was used for data synthesis and Egger test and *I*^2^ to assess study bias and heterogeneity. Data were analyzed from January to December 2022. This study followed the Preferred Reporting Items for Systematic Reviews and Meta-analyses (PRISMA) guideline.

**Main Outcomes and Measures:**

Psychiatric and reproductive system disorders.

**Results:**

A total of 1197 records were identified, of which 50 met the inclusion criteria for qualitative and 31 for quantitative synthesis. Diagnosis of a reproductive system disorder was associated with a 2- to 3-fold increased odds of having a psychiatric disorder (lower bound odds ratio [OR], 2.00; 95% CI, 1.41-2.83; upper bound OR; 2.88; 95% CI, 2.21-3.76). The analysis focused on specific diagnoses described in the literature and found that polycystic ovary syndrome was associated with increased odds of depression (population-based studies OR, 1.71; 95% CI, 1.19-2.45; clinical studies OR, 2.58; 95% CI, 1.57-4.23) and anxiety (population-based studies OR, 1.69; 95% CI, 1.36-2.10; clinical studies OR, 2.85; 95% CI, 1.98-4.09). Chronic pelvic pain was also associated with both depression (OR, 3.91; 95% CI, 1.81-8.46) and anxiety (OR, 2.33; 95% CI, 1.33-4.08). Few studies investigated risk of other reproductive system disorders in women with psychiatric disorders, or reverse associations (risk of reproductive system disorder among women with a psychiatric diagnosis).

**Conclusions and Relevance:**

In this systematic review and meta-analysis, a high rate of reported co-occurrence between psychiatric and reproductive disorders overall was observed. However, data for many disorder pairs were limited. The available literature focused overwhelmingly on affective disorders in polycystic ovary syndrome, overlooking a substantial portion of disease overlap. As such, the associations between the majority of mental health outcomes and conditions of the female reproductive system are largely unknown.

## Key Points

**Question:**

Is there an association between reproductive system disorders and psychiatric disorders in women?

**Findings:**

This review of 50 studies and meta-analysis of 31 studies identified an approximately 2- to 3-fold increased odds of having a psychiatric disorder in women with reproductive system disorders.

**Meaning:**

Despite the high rate of comorbidity for psychiatric and reproductive system disorders found in this study, data are too limited to suggest a shared cause.

## Teaser

This systematic review and meta-analysis evaluates the association between mental health and reproductive system disorders in women.

## Introduction

Reproductive system and mental health disorders represent common morbidities among women of reproductive age,^1,2,3^ and the rate of co-occurrence of these disorders is high. Although the causes of this comorbidity remain largely unknown, possible explanations include external factors such as psychotropic medications interfering with reproductive function,^4^ psychosocial factors such as reproductive system disorders affecting relationships, and overall quality of life^5^ and stress impacting menstrual cycles and reproductive function.^6^ Additionally, the disease overlap may occur due to a partially shared genetic cause.^7,8^

Compelling evidence in support of the interdependence between psychiatric and reproductive system functions comes from studies demonstrating (1) the sexually dimorphic character of many psychiatric and neurodevelopmental disorders, including differential symptoms,^9,10^ age of onset,^9,10,11^ and prevalence^12,13,14^; (2) fluctuation in severity of psychiatric morbidities during the menstrual cycle^15,16^; (3) perinatal and perimenopausal onset of several psychiatric disorders^17,18^; and (4) reduced fecundity in individuals with mental illness.^19^

To address the research gap on comorbidity between psychiatric and reproductive system disorders, our objectives were to (1) systematically review the literature on associations between psychiatric and reproductive system disorders in women of reproductive age; (2) perform a meta-analysis on risk of psychiatric morbidity associated with disorders of the reproductive system, and vice versa; and (3) perform meta-analyses as in objective 2 but stratified by specific psychiatric-reproductive system disorder pairs.

## Methods

The protocol for this study was preregistered at PROSPERO. We followed Preferred Reporting Items for Systematic Reviews and Meta-analyses (PRISMA) reporting guidelines^20^ with reference to selection and synthesis of the available evidence.

### Participants

Participants were women of reproductive age. Whenever information on puberty or menopause onset was missing, we defined that as women aged 13 to 55 years.^21,22^

### Interventions and Outcomes

 We included studies where either psychiatric or reproductive system diagnosis were used as case or control ascertainment criterion, and the other diagnostic category as outcome. To address potential confounding, we excluded psychiatric and reproductive disorders triggered by life events (eg, trauma, infection, or surgery). The range of the diagnoses included in each of these groups is presented subsequently and in eTable 1 in Supplement 1.

For psychiatric diagnoses, we included diagnoses of psychotic (F20-F29), affective (F30-39), anxiety (F40-F48), behavioral syndromes (F50-F59), personality (F60-F69), neurodevelopmental and other early onset psychiatric disorders (F70-F99), as well as the respective diagnoses made using *International Classification of Diseases, Eighth Revision (ICD-9)*, *International Classification of Diseases, Ninth Revision (ICD-10)*, *Diagnostic and Statistical Manual of Mental Disorders* (Fourth Edition) (*DSM-IV*), and *Diagnostic and Statistical Manual of Mental Disorders* (Fifth Edition) (*DSM-5*) criteria (see eTable 1 in Supplement 1). We did not consider psychiatric disorders that arose due to substance use, physical trauma, sexual dysfunction, childbirth, infertility, and use of artificial reproduction techniques.

For reproductive system diagnoses, we included inflammatory diseases of female pelvic organs (N70-N77), noninflammatory disorders of female genital tract (N80-N94), and ovarian dysfunction (E28), as well as respective diagnoses made using *ICD-9* and *ICD-10* criteria (see eTable 1 in Supplement 1). We did not consider reproductive conditions that arose due to distinct environmental causes including sexually transmitted infection, surgery, or medication.

### Comparisons

We made 4 comparisons. First, we compared lifetime risk of any psychiatric disorder among women with lifetime diagnosis of any reproductive system disorder; second, lifetime risk of specific psychiatric disorders among women with specific reproductive system disorders; third, lifetime risk of any reproductive system disorder among women with lifetime diagnosis of any psychiatric disorder; fourth, lifetime risk of specific reproductive system disorders among women with specific psychiatric disorders

### Study Characteristics

We included observational studies (case-control and population-based cross-sectional) published between January 1980 and December 2019 that were peer-reviewed and published by December 2019. We excluded studies conducted in or after 2020 due to the unknown impact of the COVID-19 pandemic on the relationship between mental health and reproductive outcomes.

### Information Sources, Search Strategy and Record Management

The search for relevant literature was conducted using Distiller SR software (Evidence Partners) and included the records listed in PubMed. The search words were selected using the list of the relevant *ICD* and *DSM* diagnoses (eTable 1 in Supplement 1) and combined using Boolean logic principles (eTable 2 in Supplement 1).

All references were checked for duplicates, stored, and managed using Distiller SR software. Two authors (N.Z. and A.B., N.Z. and E.L., or N.Z. and M.J.) independently screened each reference over 3 filtering steps: (1) rapid title screening, (2) abstract screening, and (3) selection of articles for the meta-analysis and data extraction. At each step, consensus regarding article inclusion and exclusion was established between both authors.

Data extraction was done using prespecified forms, including information on the study characteristics (authors, outcomes, interventions, and sample size) and results (proportion of exposed cases and controls).

### Statistical Analysis

To synthesize the data, we used a random-effects model using the reciprocal of the estimated variance, allowing for combining effect size estimates without individual-level data (metafor package in R, version 4.0.4; R Project for Statistical Computing^23^). From each study, we extracted crude (unadjusted) odds ratios (ORs) and their 95% CIs. Statistical significance was determined at α = .05.

We removed all data lines with fewer than 5 cases or controls with or without the outcome to avoid sparse data bias.^24,25^ To evaluate study heterogeneity and potential publication bias, we computed *I*^2^, inspected funnel plots, and applied an Egger test. Furthermore, we pooled studies according to sampling characteristics (ie, population-based, clinical, and clinical after exclusion of data lines with <10 cases or controls with or without the outcome).

Studies varied largely in diagnosis ascertainment (eg, dichotomous [yes or no] diagnoses vs ordinal scales of mild, moderate, and severe symptom levels). To avoid inflating results by considering all levels of ordinal scales as individual outcomes, symptom levels were summarized as single dichotomous yes or no variables and included in the analyses as single exposure-outcome associations. Some studies analyzed multiple outcomes (eg, depression and bipolar disorder [BD] in women with polycystic ovary syndrome [PCOS]) without stating the rate of comorbidities between them. To avoid inflating the overall pooled estimates by counting the same (comorbid) individual more than once, we calculated lower and upper bounds of the association by including, respectively, only the lowest and highest effect size per primary outcome per study. For example, if 1 study assessed depression (highest effect size), anxiety (middle effect size), and schizophrenia (lowest effect size) in PCOS, we only included depression for the upper bound and only schizophrenia for the lower bound estimates. Data were analyzed from January to December 2022.

## Results

**Figure 1.**
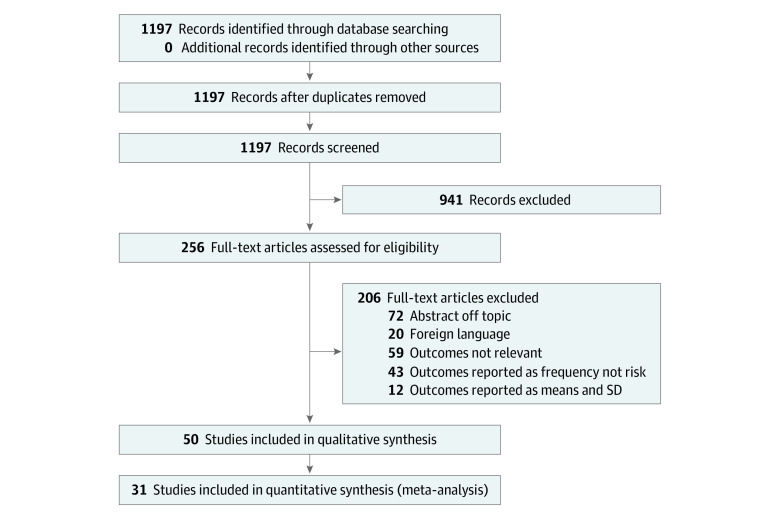
Flowchart of Included Articles PRISMA diagram of study selection.

Our search identified 1197 records, 50^8,26,27,28,29,30,31,32,33,34,35,36,37,38,39,40,41,42,43,44,45,46,47,48,49,50,51,52,53,54,55,56,57,58,59,60,61,62,63,64^ of which met the inclusion criteria for qualitative and 31^8,26,27,28,29,30,31,32,33,34,35,36,37,38,39,40,41,42,43,44,46,47,48,49,50,51,52,53,54^ for quantitative synthesis (Figure 1). Thirty^8,26,28,29,30,31,32,33,34,35,36,37,38,39,40,41,42,43,44,45,46,47,48,49,50,51,52,53,54,65^ of the latter ascertained individuals according to reproductive diagnosis status (affected or unaffected) and evaluated rate of psychiatric morbidity within those groups. Only 2 studies^26,27^ performed the opposite, that is, ascertained study samples according to psychiatric diagnosis status, 1 of which explored associations in both directions.^26^ Study characteristics are displayed in eTable 3 and further details on studies are displayed in eTable 4 in Supplement 1. Overall, we found approximately 2 to 3 times overall increased odds of psychiatric disorders in women with reproductive system disorders. The majority of the identified studies were fairly small (median [IQR] data cell size, 58.5 [27-901]).

### Risk of Any Psychiatric Diagnosis Among Patients with Any Reproductive System Diagnosis

Diagnosis of a reproductive system disorder was associated with increased odds of a psychiatric diagnosis (lower bound OR, 2.00; 95% CI, 1.41-2.83; upper bound OR; 2.88; 95% CI, 2.21-3.76; note that the upper bound is likely inflated due to inclusion of multiple estimates per study). Substantial heterogeneity between the studies was evidenced by the high *I*^2^ values (*I*^2^ = 94.7 and *I*^2^ = 96.3, respectively). An Egger test showed no evidence of small study bias (β̂_0_ = −31.30; SE, 23.3; *t* = −1.34; *P* = .19). However, the funnel plot revealed asymmetry and an abundance of studies lying outside of the expected 95% CI, suggesting potential publication bias (eFigure in Supplement 1).

Following exclusion of data lines with fewer than 10 cases or controls with or without the outcome, we observed a considerable decrease in estimates (lower bound OR, 1.42; 95% CI, 0.94-2.14; upper bound OR, 2.41; 95% CI, 1.78-3.26), with no effect on the measures of heterogeneity.

### Risk of Any Reproductive System Diagnosis in Patients With Any Psychiatric Diagnosis

The paucity of literature precluded pooling of estimates. In the 2 included studies, there was no association between having BD and a menstrual cycle of less than 25 days^27^; however, women with autism spectrum condition had statistically significantly increased odds of having PCOS (Rotterdam criteria) compared with women without autism spectrum condition (OR = 2.33; 95% CI, 1.76-3.08).^26^

### Pairs of Reproductive System and Psychiatric Disorders

**Figure 2.**
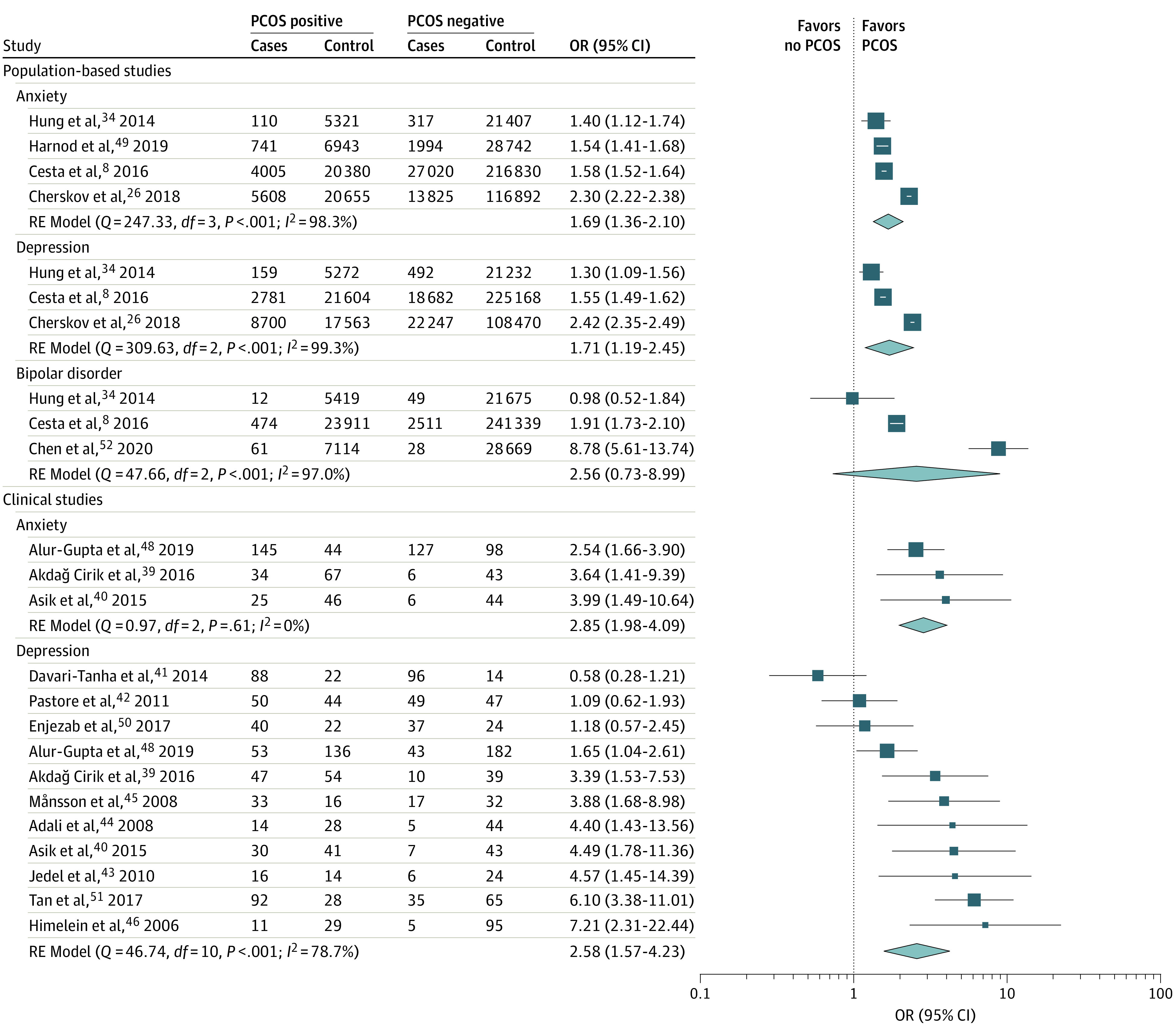
Meta-analysis of Studies on Affective Disorders in Women With Polycystic Ovary Syndrome (PCOS) Forest plots displaying odds of affective disorders in women with PCOS. Studies are grouped by study population type. An odds ratio (OR) of more than 1 indicates increased odds of each respective affective disorder in women with PCOS compared with women without PCOS.

**Figure 3.**
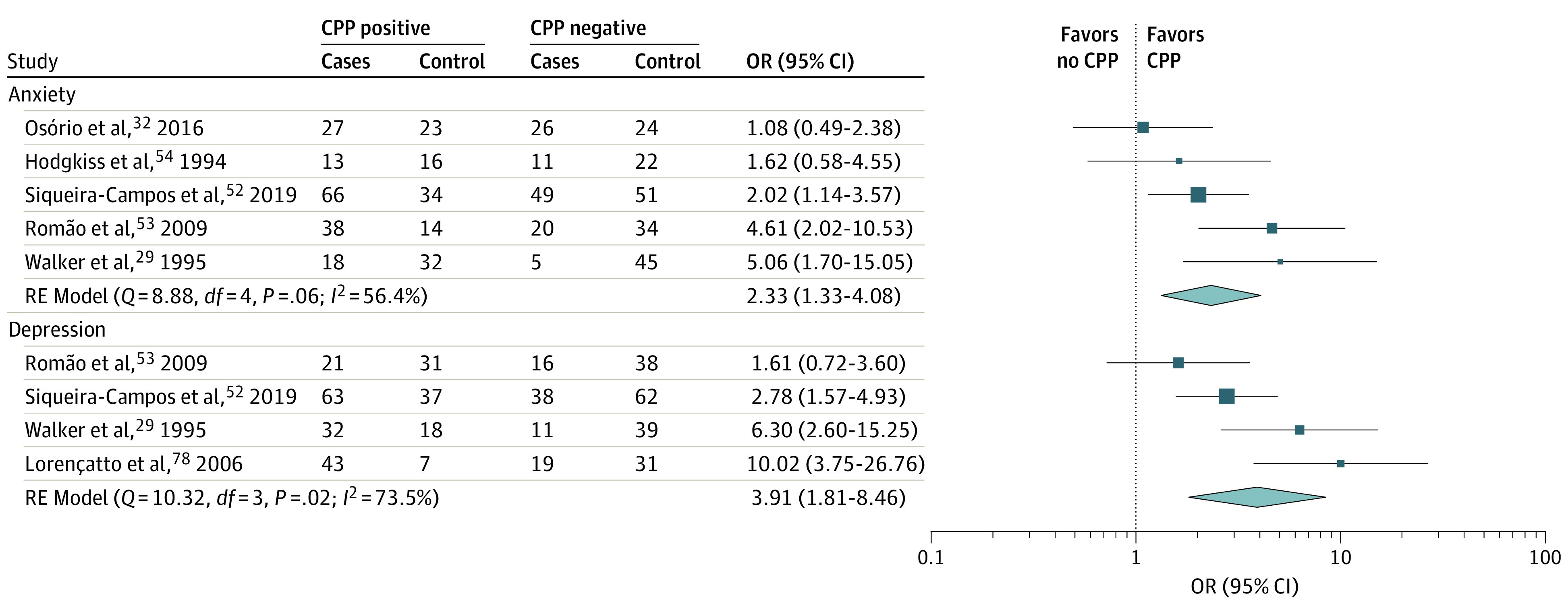
Meta-analysis of Studies on Affective Disorders in Women With Chronic Pelvic Pain (CPP) Forest plots displaying odds of affective disorders in women with CPP. An odds ratio (OR) of more than 1 indicates increased odds of each respective affective disorder in women with CPP compared with women without CPP.

**Figure 4.**
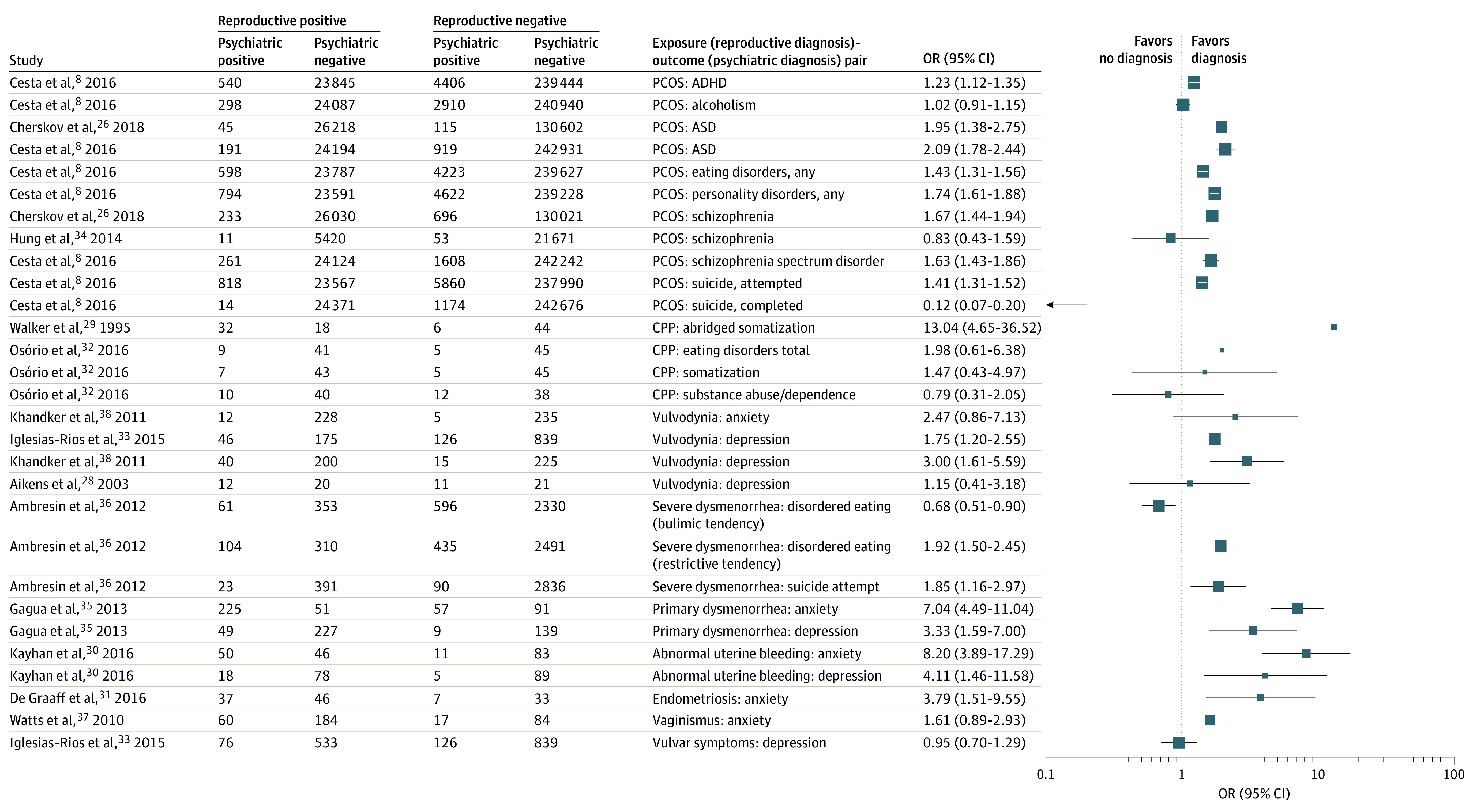
Summary of Associations Between Other Reproductive System Disorders and Psychiatric Disorders Forest plots displaying odds of psychiatric disorders in women with reproductive system disorders. An odds ratio (OR) of more than 1 indicates increased odds of each respective psychiatric disorder in women with reproductive system disorders compared with women without reproductive system disorders. ADHD indicates attention-deficit/hyperactivity disorder; ASD, autism spectrum disorder; CPP, chronic pelvic pain; PCOS, polycystic ovary syndrome.

For most diagnosis pairs, we observed positive associations. Evidence regarding the most commonly studied comorbidities, such as PCOS-affective disorders and chronic pelvic pain (CPP)-affective disorders, is presented in detail in a later section and in Figure 2 and Figure 3. Evidence regarding other pairs of comorbidities is presented in Figure 4.^8,26,28,29,30,31,32,33,34,35,36,37,38^

### PCOS and Affective Disorders

#### Meta-analysis

Our systematic review included 23 articles^8,26,34,39,40,41,42,43,44,45,46,47,48,49,50,51,66,67,68,69,70,71,72^ investigating the overlap between PCOS and the affective disorders: depression, anxiety, and BD. Of these, 16^8,26,34,39,40,41,42,43,44,45,46,47,48,49,50,51^ were eligible for inclusion in quantitative analyses (Figure 2). A total of 438 128 individuals were included in depression studies, 475 413 in anxiety studies, and 331 262 in BD studies.

In population-based studies, the combined odds of depression, anxiety disorders, and BD in women with PCOS were 1.71 (95% CI, 1.19-2.45; *I*^2^ = 99.3%), 1.69 (95% CI, 1.36-2.10; *I*^2^ = 98.4%), and 2.56 (95% CI, 0.73-8.99; *I*^2^ = 97.0%), respectively, compared with women without PCOS. As evidenced by high *I*^2^ values, heterogeneity between studies was substantial.

In clinical studies, the combined odds of depression and anxiety disorders increased to 2.58 (95% CI, 1.57-4.23; *I*^2^ = 78.7%) and 2.85 (95% CI, 1.98-4.09; *I*^2^ = 0.0%), respectively (no clinical studies on BD were included). After exclusion of data lines in clinical studies with fewer than 10 cases or controls with or without the outcome, only depression studies were available for pooling, for which the combined odds were 1.92 (95% CI, 1.04-3.54; *I*^2^ = 84.0%). Although heterogeneity between depression studies remained substantial, it was not present between anxiety disorder studies.

Effect size estimates for depression and anxiety disorders were substantially higher in clinically ascertained samples. Still, in both population-based and clinical studies, the odds of these disorders were statistically significantly higher in women with PCOS compared with those without PCOS. Odds of BD did not differ according to PCOS status.

#### Literature Overview: Depression and Anxiety Disorders

Polycystic ovary syndrome affects 5% to 10% of women of reproductive age.^73,74^ Some of the putative reasons underlying the increased risk of depression and anxiety in patients with PCOS include physical manifestations (eg, infertility, metabolic syndromes, obesity, acne, hirsutism^8,39,48,73^) and their impact on body satisfaction, adverse effects of medications used to manage PCOS on mood (eg, metformin and oral contraceptives^75^), the role of androgens,^8^ and/or shared underlying genetic factors.^8,72^

Importantly, ascertainment strategies differed substantially across studies, potentially undermining the strength of reported associations and limiting generalizability of findings. Few studies stated PCOS ascertainment criteria (National Institutes of Health vs Rotterdam); some^34,43,49,51^ required that cases have no psychiatric history before the onset of PCOS, and some^45,51,68,69^ required controls to have regular menstrual cycles or to have no history of mental health problems.^43,44^

Critically, only in a few studies^42,43,48,51,67^ were PCOS cases and controls matched on factors such as body mass index (BMI) infertility, or hirsutism. Among the studies with matched BMI, all^43,48,51^ but one^42^ still reported higher rates of depression and anxiety in PCOS cases. Similarly, rates of depression in PCOS were higher irrespective of infertility status.^46^ In other studies, PCOS cases with affective disorders were more likely to have high BMI and experience menstrual irregularity, infertility, or hirsutism.^40,44,45,51,67,70^ However, it remains unclear to what extent these health concerns are independent risk factors for affective disorders vs characteristics of a more severe PCOS phenotype overall. Currently, the emerging consensus suggests that high BMI and infertility may exacerbate, but do not fully explain, affective symptoms in PCOS.

Few studies collected biochemical measures. One study demonstrated an association between the risk of free androgen and risk of affective disorders in PCOS,^45^ while other studies^39,42,50^ found no evidence for such an association. Additionally, there were no differences in any of the inflammatory markers between cases of PCOS with and without depression.^69^

Addressing the possibility of shared genetics, Cesta et al (1) compared the risk of affective disorders in cases of PCOS and their unaffected siblings,^8^ and (2) performed a twin analysis to estimate the genetic and nongenetic underpinnings of the co-occurrence of these disorders.^72^ Both studies suggested that the comorbidity is at least in part due to shared genetic factors, as demonstrated by an increased risk of depression in sisters of women with PCOS (OR, 1.11; 95% CI, 1.02-1.21) compared with population controls, and a high fraction (63%) of comorbidity between PCOS and depression attributable to common genetic factors in twins.

#### Literature Overview: BD

Among the 6 BD studies,^8,34,41,45,47,66^ 2 reported no evidence for a significantly increased risk in patients with PCOS^34^ (note that 1 study measured “any manic or hypomanic episode” and not necessarily BD^45^). One study^8^ assessed diagnosis overlap irrespective of the temporal order, while 2 population-based studies^34,47^ assessed only psychiatric outcomes occurring after PCOS diagnosis. Two clinical studies^41,66^ measured psychopathology in PCOS cases and controls. Notably, study precision may have been impacted by the relative rarity of BD in the population (lifetime prevalence of approximately 1% in the US^76,77^).

None of the studies differentiated between BD types 1 and 2. Only Chen et al^47^ considered the role of medication in mediating this comorbidity, and found a reduced risk of BD in patients with PCOS treated with metformin and hormone therapy. Conversely, BD treatment with valproate has been suggested to induce PCOS or PCOS-related phenotypes (eg, menstrual abnormalities, elevated glucose^78^). BD risk was not significantly elevated in either male or female siblings of patients with PCOS in Cesta et al,^8^ providing no evidence of genetic association between PCOS and BD.

### CPP and Affective Disorders

#### Meta-analysis

We included 8 articles exploring CPP and affective disorders in our systematic review.^29,32,52,53,54,65,79,80^ Of these, 6 were included in the quantitative analysis (Figure 3) (the first of the excluded studies^80^ was a nested sample of another,^29^ and the second was too small). A total of 506 individuals were included in depression studies and 568 individuals in anxiety studies.

Among women with CPP, the combined odds of depression and anxiety-related disorders were 3.91 (95% CI, 1.81-8.46; *I*^2^ = 73.5%) and 2.33 (95% CI, 1.33-4.08; *I*^2^ = 56.4%), respectively. Heterogeneity between studies was moderate, and this was not associated with data lines with fewer than 10 cases or controls with or without the outcome. After exclusion of those studies, the pooled odds ratios for depression and anxiety were 2.97 (95% CI, 1.47-6.01; *I*^2^ = 62.8%) and 2.02 (95% CI, 1.13-3.62; *I*^2^ = 54.9%), respectively.

Effect size estimates for depression and anxiety disorders were higher before removal of data lines with fewer than 10 cases or controls with or without the outcome. Still, in both instances, the odds of these disorders were statistically significantly higher in women with CPP compared with those without CPP.

#### Literature Overview

CPP affects 1 in 7 women in the US.^81^ All studies found statistically significantly higher levels of depression in those with CPP compared with controls.^29,32,52,53,54,65,79,80^ Although anxiety was also more common in CPP groups,^29,52,53,79,80^ this difference was not always statistically significant.^32,54^ Of note, the causes of CPP vary considerably. Laparoscopic findings possibly underlying pelvic pain generally indicate endometriosis, pelvic adhesions affecting the genital tract and bowel, or an absence of findings.^82,83^ The cause of the pain, however, does not appear to be associated with affective disorders. In 1 study,^54^ rates of depression did not differ according to presence or absence of laparoscopic findings. Similarly, in 100 endometriosis cases, those with accompanying CPP were significantly more depressed than those without pain,^65^ suggesting that pain irrespective of underlying pathology is associated with depression. In another study,^52^ increased rates of depression and anxiety in women with CPP were not associated with intensity or duration of pain, suggesting that pain irrespective of magnitude is associated with psychiatric morbidity.

Associations between CPP and affective disorders were investigated in the context of past sexual trauma. In a pilot study, CPP and lifetime depression were associated only among victims of childhood sexual abuse, and 12 of 16 CPP cases with lifetime depression had their first episode of major depression before onset of CPP.^80^ In a follow-up study, the rate of childhood sexual trauma was comparable with that of the general population in controls (2 of 50) but substantial in the CPP group (12 of 50).^29^ However, this association was not present in another similar study.^79^ In a pooled group of CPP cases and controls, CPP, physical abuse, and sexual abuse were each independently associated with depression and anxiety; however, rate of sexual abuse did not differ between cases and controls.^52^ Another study found higher levels of early emotional traumas (including sexual events) in CPP cases compared with controls, but this difference was not statistically significant.^32^

### Other Pairs of Diagnoses

We identified 13 articles^8,26,28,30,31,32,33,34,35,36,37,38,84^ exploring associations outside of PCOS, CPP, and affective disorders (Figure 4). The lack of overlap in exposure-outcome pairs precluded pooling estimates; however, nearly all diagnosis pairs were positively associated.

## Discussion

This systematic review and meta-analsysis explored evidence for the overlap between the reproductive system and psychiatric disorders. We found approximately 2 to 3 times overall increased odds of psychiatric disorders in women with reproductive system disorders. Additionally, in disorder pairs well-represented in the literature, odds of affective disorders in women with PCOS and CPP were approximately 1.7 to nearly 4 times those of women without those disorders.

Notably, the causes of both PCOS and CPP are diverse, and likely so are the mechanisms underlying their associations with psychiatric outcomes. PCOS often involves symptoms such as infertility, hormonal imbalance, hirsutism, and medication use, which can themselves spur adverse mental health outcomes. Nevertheless, as highlighted by our literature search, these factors do not seem to fully explain the observed association with affective disorders, leaving a possibility that genetic factors may also be associated with disease overlap. In support of this notion, in an epidemiologic study on all live births from 1996 to 2014 in Finland, maternal PCOS was associated with increased risk of a wide variety of psychiatric disorders in offspring, including mood, anxiety and autism spectrum disorders.^85^ Our finding on the positive association between CPP and depressive symptoms is well aligned with the evidence suggesting all forms of chronic pain are associated with depression,^86^ although we cannot at this point discern whether the CPP-depression association is distinct.

The strengths of the current study include a preregistered protocol, a large volume of screened studies, independent validation of included studies by 2 reviewers, and the collaboration of an interdisciplinary team of epidemiologists, psychiatrists, and maternal-fetal medicine specialists. Additionally, analyses were performed with careful avoidance of sample overlap so as not to inflate results.

### Limitations

This study had limitations. The narrow scope of research outside of PCOS or CPP and affective disorders, the disproportionately smaller volume of literature on reproductive outcomes among women with psychiatric disorders compared with vice versa, and shortage of genetic studies precluded exploring these comorbidities as causally associated disease classes. Lack of extensive demographic and clinical data further compromised investigations into the co-occurrence of these disorders.

The overrepresentation of small, clinically ascertained samples (median [IQR] data cell size, 58.5 [27-901]) contributed to a high degree of study heterogeneity. This was not mitigated through excluding studies with data cell sizes of fewer than 10, suggesting that, aside from the heterogeneity in mechanisms underlying the diverse set of comorbidities, study designs and analytical decisions likely compromised the evidence. Furthermore, methodological issues included inconsistent specification of past vs concurrent disorders, lack of professional determinations of diagnoses, and rare consideration of temporal relationships between disease onsets.

## Conclusions

In this systematic review and meta-analysis study on associations between psychiatric and reproductive system disorders, we identified increased odds of psychiatric disorders in women with reproductive system disorders. Further investigations into these associations are needed to understand whether these disorders are causally associated. To improve the quality of the evidence, with implications for clinical care, future studies should place greater emphasis on the collection of accurate mental health data in reproductive health settings, and deeper inquiry into somatic concerns, reproductive disorders, and menstrual status in psychiatric settings.

## Notes

Supplement 1. **eTable 1. **Diagnoses Considered in the Systematic Review and Meta-analysis **eTable 2. **Boolean Logic Used to Identify Articles **eTable 3. **Characteristics of All Included Studies **eFigure. **Funnel Plot of Included Studies on Psychiatric Outcome According to Reproductive System Disorder Status **eTable 4. **Newcastle-Ottawa Quality Assessment for Case-Control Studies

Supplement 2.
Data Sharing Statement
