# Supplementary material for: Association Between Mental Health and Reproductive System Disorders in Women: A Systematic Review and Meta-analysis
Source: JAMA Netw Open. 2023 Apr 18;6(4):e238685. doi: 10.1001/jamanetworkopen.2023.8685 (PMC10114079; doi:10.1001/jamanetworkopen.2023.8685)
Supplement: Supplement 1. — eTable 1. Diagnoses Considered in the Systematic Review and Meta-analysis eTable 2. Boolean Logic Used to Identify Articles eTable 3. Characteristics of All Included Studies eFigure. Funnel Plot of Included Studies on Psychiatric Outcome According to Reproductive System Disorder Status eTable 4. Newcastle-Ottawa Quality Assessment for Case-Control Studies [file jamanetwopen-e238685-s001.pdf]

Supplemental Online Content

Zaks N, Batuure A, Lin E, et al. Association between mental health and reproductive system disorders in women: a systematic review and meta-analysis. *JAMA Netw Open*. 2023;4(6):e238685.
doi:10.1001/jamanetworkopen.2023.8685

eTable 1. Diagnoses Considered in the Systematic Review and Meta-analysis
eTable 2. Boolean Logic Used to Identify Articles
eTable 3. Characteristics of All Included Studies
eTable 4. Newcastle-Ottawa Quality Assessment for Case-Control Studies
eFigure. Funnel Plot of Included Studies on Psychiatric Outcome According to Reproductive System Disorder Status

This supplemental material has been provided by the authors to give readers additional information about their work.

© 2023 Zaks N et al. *JAMA Network Open.*

eTable 1. Diagnoses Considered in the Systematic Review and Meta-analysis

	Psychiatric (1a)	***Reproductive (1b)***
ICD-8	295 Schizophrenia<br>296 Affective psychoses<br>297 Paranoid states<br>298 Other psychoses<br>299 Unspecified psychosis<br>300 Neuroses<br>301 Personality disorders<br>308 Behaviour disorders of childhood<br>309 Mental disorders not specified as psychotic associated with physical conditions<br>310 Borderline mental retardation<br>311 Mild mental retardation<br>312 Moderate mental retardation<br>313 Severe mental retardation<br>314 Profound mental retardation<br>315 Unspecified mental retardation	256 Ovarian dysfunction<br>**612 Acute salpingitis and oophoritis<br>**613 Chronic salpingitis and oophoritis<br>**614 Salpingitis and oophoritis, unqualified<br>615 Other diseases of ovary and Fallopian tube<br>616 Diseases of parametrium and pelvic peritoneum (female)<br>**620 Infective diseases of cervix uteri<br>621 Other diseases of cervix<br>**622 Infective diseases of uterus (except cervix), vagina and vulva<br>**623 Uterovaginal prolapse<br>624 Malposition of uterus<br>625 Other diseases of uterus<br>626 Disorders of menstruation<br>629 Other diseases of female genital organs<br>**630 Infections of genital tract during pregnancy
ICD-9	295 Schizophrenic disorders<br>296 Episodic mood disorders<br>297 Delusional disorders<br>298 Other nonorganic psychoses<br>299 Pervasive developmental disorders<br>300 Anxiety, dissociative and somatoform disorders<br>301 Personality disorders<br>307 Special symptoms or syndromes not elsewhere classified<br>**308 Acute reaction to stress<br>**309 Adjustment reaction<br>311 Depressive disorder, not elsewhere classified<br>312 Disturbance of conduct not elsewhere classified<br>313 Disturbance of emotions specific to childhood and adolescence<br>314 Hyperkinetic syndrome of childhood<br>315 Specific delays in development<br>317 Mild intellectual disabilities<br>318 Other intellectual disabilities<br>319 Unspecified intellectual disabilities	256 Ovarian dysfunction<br>**618 Genital prolapse<br>**619 Fistula involving female genital tract<br>620 Noninflammatory disorders of ovary fallopian tube and broad ligament<br>621 Disorders of uterus not elsewhere classified<br>622 Noninflammatory disorders of cervix<br>623 Noninflammatory disorders of vagina<br>624 Noninflammatory disorders of vulva and perineum<br>625 Pain and other symptoms associated with female genital organs<br>626 Disorders of menstruation and other abnormal bleeding from female genital tract<br>629 Other disorders of female genital organs
ICD-10	F20 Schizophrenia<br>F21 Schizotypal disorder<br>F22 Delusional disorders<br>F23 Brief psychotic disorder<br>F24 Shared psychotic disorder<br>F25 Schizoaffective disorders<br>F28 Other psychotic disorder not due to a substance or known physiological condition<br>F29 Unspecified psychosis not due to a substance or known physiological condition<br>F30 Manic episode<br>F31 Bipolar disorder<br>F32 Major depressive disorder, single episode<br>F33 Major depressive disorder, recurrent<br>F34 Persistent mood [affective] disorders<br>F39 Unspecified mood [affective] disorder<br>F40 Phobic anxiety disorders<br>F41 Other anxiety disorders<br>F42 Obsessive-compulsive disorder<br>**F43 Reaction to severe stress, and adjustment disorders<br>F44 Dissociative and conversion disorders	E28 Ovarian dysfunction<br>**N70 Salpingitis and oophoritis<br>N71 Inflammatory disease of uterus, except cervix<br>N72 Inflammatory disease of cervix uteri<br>N73 Other female pelvic inflammatory diseases<br>**N74 Female pelvic inflammatory disorders in diseases classified elsewhere<br>N75 Diseases of Bartholin's gland<br>N76 Other inflammation of vagina and vulva<br>**N77 Vulvovaginal ulceration and inflammation in diseases classified elsewhere<br>N80 Endometriosis<br>**N81 Female genital prolapse<br>**N82 Fistulae involving female genital tract<br>N83 Noninflammatory disorders of ovary, fallopian tube and broad ligament<br>N84 Polyp of female genital tract<br>N85 Other noninflammatory disorders of uterus, except cervix<br>N86 Erosion and ectropion of cervix uteri<br>**N87 Dysplasia of cervix uteri

© 2023 Zaks N et al. *JAMA Network Open.*

	F45 Somatoform disorders<br>F48 Other nonpsychotic mental disorders<br>F50 Eating disorders<br>F51 Sleep disorders not due to a substance or known physiological condition<br>F54 Psychological and behavioral factors associated with disorders or diseases classified elsewhere<br>F59 Unspecified behavioral syndromes associated with physiological disturbances and physical factors<br>F60 Specific personality disorders<br>F63 Impulse disorders<br>F68 Other disorders of adult personality and behavior<br>F69 Unspecified disorder of adult personality and behavior<br>F70 Mild intellectual disabilities<br>F71 Moderate intellectual disabilities<br>F72 Severe intellectual disabilities<br>F73 Profound intellectual disabilities<br>F78 Other intellectual disabilities<br>F79 Unspecified intellectual disabilities<br>F80 Specific developmental disorders of speech and language<br>F81 Specific developmental disorders of scholastic skills<br>F82 Specific developmental disorder of motor function<br>F84 Pervasive developmental disorders<br>F88 Other disorders of psychological development<br>F89 Unspecified disorder of psychological development<br>F90 Attention-deficit hyperactivity disorders<br>F91 Conduct disorders<br>F93 Emotional disorders with onset specific to childhood<br>F94 Disorders of social functioning with onset specific to childhood and adolescence<br>F95 Tic disorder<br>F98 Other behavioral and emotional disorders with onset usually occurring in childhood and adolescence<br>F99 Mental disorder, not otherwise specified	N88 Other noninflammatory disorders of cervix uteri<br>N89 Other noninflammatory disorders of vagina<br>N90 Other noninflammatory disorders of vulva and perineum<br>N91 Absent, scanty and rare menstruation<br>N92 Excessive, frequent and irregular menstruation<br>N93 Other abnormal uterine and vaginal bleeding<br>N94 Pain and other conditions associated with female genital organs and menstrual cycle
DSM-IV	As in ICD-9	
DSM-5	As in ICD-10	

***Diagnoses excluded from original protocol.*

© 2023 Zaks N et al. *JAMA Network Open.*

eTable 2. Boolean Logic Used to Identify Articles

((Reproductive) OR (genitourinary) OR (urogenital) OR (genital) OR (obstetrical) OR (female genital tract) OR (cervix) OR (cervical) OR (pubocervical) OR(cervicitis) OR (endocervicitis) OR (vulva) OR (vulvar) OR (vulvitis) OR (ovary) OR (ovarian) OR (oophoritis) OR (corpus luteum) OR (uterus) OR (uterine) OR (intrauterine) OR (corpus uteri) OR (vagina) OR (vaginal) OR (vaginitis) OR (vulvovaginal) OR (vulvovaginitis) OR (uterovaginal) OR (rectovaginal) OR (pelvis) OR (pelvic) OR (foetopelvic) OR (parametrium) OR (parametritis) OR (pelvic cellulitis) OR (pelvic peritonitis) OR (fallopian tube) OR (salpingitis) OR (Bartholin's gland) OR (Fallopian tube) OR (greater vestibular gland) OR (broad ligament) OR (Müllerian ducts) OR (hymen) OR (hymenal) OR (clitoris) OR (labia) OR (hematometra) OR (perineum) OR (menstruation) OR (menstrual) OR (leukorrhea) OR (amenorrhea) OR (oligomenorrhea) OR (dysmenorrhea) OR (vaginal bleeding) OR (infertility) OR (sterility) OR (pregnancy) OR (cystocele) OR (urethrocele) OR (rectocele) OR (perineocele) OR (dyspareunia) OR (vaginismus) OR (mittelschmerz) OR (vulvodynia) OR (metrorrhagia) OR (canal of Nuck) OR (hematosalpinx) OR (hematocolpos) OR (didelphys) OR ((cyst) AND (follicular))) AND
((psychiatric) OR (schizophrenia) OR (psychosis) OR (paranoia) OR (paranoid) OR (paranoidal) OR (schizotypal) OR (delusional) OR (schizoaffective) OR (paraphrenia) OR (nonpsychotic) OR (psychotic) OR (reactive confusion) OR (manic) OR (Bipolar AND ((I) OR (II)) OR (depression) OR (depressive) OR (dysthymia) OR (neurasthenia) OR (phobic) OR (anxiety) OR (anxious) OR (overanxious) OR (neurosis) OR (neurotic) OR (hysteria) OR (obsessive-compulsive) OR (panic) OR (hypochondrias) OR (hypochondriasis) OR (posttraumatic) OR ((disturbance) AND ((emotions) OR (consciousness) OR (conduct))) OR (reaction to stress) OR (pathological gambling) OR (kleptomania) OR (pyromania) OR (factitious illness) OR (intellectual disability) OR ((developmental) AND ((disorder) OR (delay)) OR (autism) OR (autistic) OR (ADHD) OR (Attention deficit) OR (hyperkinetic syndrome) OR (hyperkinesis) OR ((Disorder) AND ((behavioral) OR (affective) OR (bipolar) OR (mood) OR (dysthymic) OR (conduct) OR (impulse control) OR (emotional) OR (tic) OR (language) OR (introverted) OR (disintegrative) OR (eating) OR (sleep) OR (personality) OR (somatoform) OR (adjustment) OR (dissociative) OR (depersonalization) OR (emancipation) OR (explosive) OR (fluency))) OR (sensitivity shyness) OR (social withdrawal) OR (selective mutism) OR (alexia) OR (dyslexia) OR (Intellectual disability)) AND (Reproductive[Title] OR genitourinary[Title] OR urogenital[Title] OR genital[Title] OR obstetrical[Title] OR female genital tract[Title] OR cervix[Title] OR cervical[Title] OR pubocervical[Title] OR cervicitis[Title] OR endocervicitis[Title] OR vulva[Title] OR vulvar[Title] OR vulvitis[Title] OR ovary[Title] OR ovarian[Title] OR oophoritis[Title] OR corpus luteum[Title] OR uterus[Title] OR uterine[Title] OR intrauterine[Title] OR corpus uteri[Title] OR vagina[Title] OR vaginal[Title] OR vaginitis[Title] OR vulvovaginal[Title] OR vulvovaginitis[Title] OR uterovaginal[Title] OR rectovaginal[Title] OR pelvis[Title] OR pelvic[Title] OR foetopelvic[Title] OR parametrium[Title] OR parametritis[Title] OR pelvic cellulitis[Title] OR pelvic peritonitis[Title] OR fallopian tube[Title] OR salpingitis[Title] OR Bartholin's gland[Title] OR Fallopian tube[Title] OR greater vestibular gland[Title] OR broad ligament[Title] OR Müllerian ducts[Title] OR hymen[Title] OR hymenal[Title] OR clitoris[Title] OR labia[Title] OR hematometra[Title] OR perineum[Title] OR menstruation[Title] OR menstrual[Title] OR leukorrhea[Title] OR amenorrhea[Title] OR oligomenorrhea[Title] OR dysmenorrhea[Title] OR vaginal bleeding[Title] OR infertility[Title] OR sterility[Title] OR cystocele[Title] OR urethrocele[Title] OR rectocele[Title] OR perineocele[Title] OR dyspareunia[Title] OR vaginismus[Title] OR mittelschmerz[Title] OR vulvodynia[Title] OR metrorrhagia[Title] OR canal of Nuck[Title] OR hematosalpinx[Title] OR hematocolpos[Title] OR didelphys[Title] OR "Follicular cyst"[Title]) AND (psychiatric[Title] OR schizophrenia[Title] OR psychosis[Title] OR paranoia[Title] OR paranoid[Title] OR paranoidal[Title] OR schizotypal[Title] OR delusional[Title] OR schizoaffective[Title] OR paraphrenia[Title] OR nonpsychotic[Title] OR psychotic[Title] OR reactive confusion[Title] OR manic[Title] OR depression[Title] OR depressive[Title] OR dysthymia[Title] OR neurasthenia[Title] OR phobic[Title] OR anxiety[Title] OR anxious[Title] OR overanxious[Title] OR neurosis[Title] OR neurotic[Title] OR hysteria[Title] OR obsessive-compulsive[Title] OR panic[Title] OR hypochondrias[Title] OR hypochondriasis[Title] OR posttraumatic[Title]

© 2023 Zaks N et al. *JAMA Network Open.*

OR (disturbance[Title] AND emotions[Title] OR consciousness[Title] OR conduct[Title]) OR reaction to stress[Title] OR "pathological gambling"[Title] OR kleptomania[Title] OR pyromania[Title] OR factitious illness[Title] OR intellectual disability[Title] OR (developmental[Title] AND (disorder[Title] OR delay[Title])) OR autism[Title] OR autistic[Title] OR ADHD[Title] OR Attention deficit[Title] OR hyperkinetic syndrome[Title] OR hyperkinesis[Title] OR (Disorder[Title] AND (behavioral[Title] OR affective[Title] OR bipolar[Title] OR mood[Title] OR dysthymic[Title] OR conduct[Title] OR impulse control[Title] OR emotional[Title] OR tic[Title] OR language[Title] OR introverted[Title] OR disintegrative[Title] OR eating[Title] OR sleep[Title] OR personality[Title] OR somatoform[Title] OR adjustment[Title] OR dissociative[Title] OR depersonalization[Title] OR emancipation[Title] OR explosive[Title] OR fluency[Title])) OR sensitivity shyness[Title] OR social withdrawal[Title] OR selective mutism[Title] OR alexia[Title] OR dyslexia[Title] OR intellectual disability[Title]) NOT rats[Title] NOT rat[Title] NOT dog[Title] NOT dogs[Title] NOT rabbits[Title] NOT rabbit[Title] NOT mice[Title] NOT stoppage[Title] NOT artificial fertilization[Title] NOT assisted reproduction[Title] NOT menopause[Title] NOT pregnancy[Title] NOT cancer[Title] NOT case study[Title] NOT case report[Title]

© 2023 Zaks N et al. *JAMA Network Open.*

eTable 3. Characteristics of All Included Studies

#	Study	Total N	Primary diagnosis	Outcome diagnosis	N cases	N controls	N outcome in cases	N outcome in controls	Justification for exclusion in quantitative synthesis
1	Chen et al.,[52] 2020	35872	PCOS	bipolar disorder	7175	28697	61	28	--
2	Alur-Gupta et al.,[53] 2019	414	PCOS	depression	189	225	53	43	--
				anxiety			145	127	--
3	Çoban et al.,[54] 2019	59	PCOS	MDD	28	31	6	1	n<5
				ADHD			2	2	n<5
				social anxiety disorder			5	1	n<5
				separation anxiety disorder			1	0	n<5
				GAD			3	0	n<5
				panic disorder			1	0	n<5
				specific phobia			2	2	n<5
				OCD			2	1	n<5
				anorexia nervosa			0	0	n<5
				bulimia nervosa			0	0	n<5
4	Harnod et al.,[55] 2019	38420	PCOS	anxiety	7684	30736	741	1994	--
5	Mazi et al., 2019*	200	pelvic floor dysfunction	depression	100	100	43	14	Primary diagnosis derives from distinct environmental etiology
6	Meng et al., 2019*	360	postpartum depression	dysmenorrhea	120	240	77	115	Primary diagnosis derives from distinct environmental etiology
				low birth weight of child			12	3	Primary diagnosis derives from distinct environmental etiology
				preterm birth of child			8	8	Primary diagnosis derives from distinct environmental etiology
7	Siqueira-Campos et al.,[65] 2019	200	CPP	depression	100	100	63	38	--
				anxiety			66	49	--
				both depression and anxiety			54	28	Outcome overlap with depression and anxiety
8	Cherskov et al.,[26] 2018	140918	PCOS phenotype	ASC	26263	130717	45	115	--
				depression			8700	22247	--
				anxiety			5608	13825	--

© 2023 Zaks N et al. *JAMA Network Open.*

				schizophrenia			233	696	--
		5826	Autism Spectrum Condition (ASC)	PCOS (Read code)	971	4855	22	55	Outcome overlap unclear; Rotterdam criteria was used to measure PCOS
				PCOS (NIH criteria)			72	150	Outcome overlap unclear; Rotterdam criteria was used to measure PCOS
				PCOS (Rotterdam criteria)			76	171	--
				anovulation			150	443	Outcome overlap unclear; Rotterdam criteria was used to measure PCOS
				hyperandrogenemia			193	604	Outcome overlap unclear; Rotterdam criteria was used to measure PCOS
				polycystic ovaries			55	75	Outcome overlap unclear; Rotterdam criteria was used to measure PCOS
9	Cesta et al.,[56] 2017	12628	PCOS	MDD (lifetime)	752	11876	255	2482	Nested sample of Cesta, 2016
10	Enjezab et al.,[57] 2017	123	PCOS	depression (mild)	62	61	17	15	[Summated into depression total]
				depression (moderate)			17	12	
				depression (severe)			6	10	
				depression total			40	37	--
11	Tan et al.,[58] 2017	220	PCOS	depression (clinical)	120	100	33	3	Cell <5
				anxiety (trait)			16	2	Cell <5
				anxiety (state)			7	1	Cell <5
				depression symptoms			92	35	--
12	Akdağ Cirik et al.,[39] 2016	150	PCOS (NIH phenotype)	depression	54	49	25	10	[Summated into PCOS total]
				anxiety			17	6	
			PCOS (non-NIH phenotype)	depression	47	49	22	10	
				anxiety			17	6	
			PCOS total	depression	101	49	47	10	--
				anxiety			34	6	--
13	Cesta et al.,[8] 2016	268235	PCOS	schizophrenia spectrum disorder	24385	243850	261	1608	--
				schizophrenia, pure			92	5061	Outcome is subset of schizophrenia spectrum disorder
				bipolar disorder			474	2511	--
				depressive disorders, any			2781	18682	--

© 2023 Zaks N et al. *JAMA Network Open.*

				severe depression			490	3390	Outcome is subset of depressive disorders, any
				anxiety disorders, any			4005	27020	--
				social phobia			269	1886	Outcome is subset of anxiety disorders, any
				OCD			314	2318	Outcome is subset of anxiety disorders, any
				eating disorders, any			598	4223	--
				anorexia			139	1504	Outcome is subset of eating disorders, any
				bulimia			179	1331	Outcome is subset of eating disorders, any
				personality disorders, any			794	4622	--
				gender identity disorders			15	744	Outcome is outside of study scope
				autism spectrum disorders			191	919	--
				autism			36	2292	Outcome is subset of autism spectrum disorders
				asperger’s syndrome			144	802	Outcome is subset of autism spectrum disorders
				ADHD			540	4406	--
				suicide, attempted			818	5860	--
				suicide, completed			14	1174	--
				alcoholism			298	2910	--
14	De Graaff et al.,[31] 2016	123	endometriosis	anxiety (mild)	83	40	16	5	[Summated into anxiety total]
				anxiety (moderate)			17	2	
				anxiety (severe)			4	0	
				anxiety total			37	7	--
				depression (mild)			8	3	[Summated into depression total]
				depression (moderate)			5	0	
				depression (severe)			2	0	
				depression total			15	3	n<5
15	Kayhan et al.,[30] 2016	190	abnormal uterine bleeding	any mood disorder	96	94	18	4	n<5
				major depression			15	4	[Summated into depression total]
				dysthymia			3	1	[Summated into depression total]
				depression total			18	5	--
				bipolar disorder			0	1	n<5

© 2023 Zaks N et al. *JAMA Network Open.*

				any anxiety disorder			50	11	--
				GAD			18	3	n<5
				panic disorder			2	1	n<5
				OCD			22	3	n<5
				specific phobia			10	4	n<5
				social anxiety disorder			2	1	n<5
				anxiety disorder NOS			9	4	n<5
				PTSD			0	0	n<5
				comorbidity of mood and anxiety disorder			17	2	n<5
16	Osório et al.,[32] 2016	100	CPP	current major depressive	50	50	14	4	n<5
				bipolar disorder			6	1	n<5
				dysthymia			1	0	n<5
				substance abuse/dependence			10	12	--
				panic			8	3	Outcome is subset of any anxiety disorder
				obsessive-compulsive			12	9	Outcome is subset of any anxiety disorder
				post-traumatic stress			3	2	Outcome is subset of any anxiety disorder
				social anxiety			10	6	Outcome is subset of any anxiety disorder
				specific phobies			12	11	Outcome is subset of any anxiety disorder
				any anxiety disorder			27	26	--
				somatization			7	5	--
				hypochondria			4	1	n<5
				anorexia			4	0	[Summated into any eating disorders total]
				bulimia			5	5	[Summated into any eating disorders total]
				eating disorders total			9	5	--
17	Shen et al., 2016*	43860	pelvic inflammatory disease (PID)	depressive disorder	21930	21930	1308	607	Primary diagnosis derives from distinct environmental etiologies
				anxiety disorder			950	482	Primary diagnosis derives from distinct environmental etiologies

© 2023 Zaks N et al. *JAMA Network Open.*

				bipolar disorder			129	49	Primary diagnosis derives from distinct environmental etiologies
				schizophrenia			61	81	Primary diagnosis derives from distinct environmental etiologies
18	Asik et al.,[40] 2015	121	PCOS	depression	71	50	30	7	--
				anxiety			25	6	--
19	Hergüner et al., 2015*	80	PCOS	ADHD (childhood)	40	40	11	2	n<5
20	Hussain et al.,[41] 2015	150	PCOS	MDD	110	40	26	3	n<5
				GAD			17	0	n<5
				OCD			7	1	n<5
				panic disorder			17	2	n<5
				suicidality			9	0	n<5
				bipolar affective disorder			3	0	n<5
				dysthymia			2	0	n<5
				agoraphobia			1	0	n<5
21	Iglesias-Rios et al.,[33] 2015	1186	vulvodynia	depression	221	965	46	126	--
				PTSD			44	92	Outcome triggered by explicit event
		1574	short-term or past vulvar symptoms	depression	609	965	76	126	--
				PTSD			93	92	Outcome triggered by explicit event
22	Davari-Tanha et al.,[42] 2014	220	PCOS	bipolar disorder	110	110	8	0	n<5
				depression			88	96	--
23	Hung et al.,[34] 2014	27155	PCOS	schizophrenia	5431	21724	11	53	--
				bipolar disorder			12	49	--
				depressive disorder			159	492	--
				anxiety disorder			110	317	--
24	Reynolds-May et al.,[27] 2014	139	bipolar disorder	history of oligomenorrhea	103	36	8	3	n<5
				history of amenorrhea			8	2	n<5
				current oligomenorrhea			9	2	n<5
				current amenorrhea			7	2	n<5
				PCOS			5	0	n<5
				central amenorrhea			22	4	n<5

© 2023 Zaks N et al. *JAMA Network Open.*

				menstrual cycle < 25 days			24	9	--
				menstrual bleeding >10 days			32	4	n<5
25	Gagua et al.,[35] 2013	424	primary dysmenorrhea	depression (moderate)	276	148	44	9	[Summated into depression total]
				depression (severe)			5	0	
				depression total			49	9	--
				anxiety (moderate, TMAS)			104	42	[Summated into anxiety total]
				anxiety (high, TMAS)			121	15	
				anxiety total			225	57	--
				anxiety (moderate, STAI)			82	62	Outcome overlap unclear; TMAS was used to measure anxiety
				anxiety (high, STAI)			190	37	Outcome overlap unclear; TMAS was used to measure anxiety
26	Ambresin et al.,[36] 2012	3340	severe dysmenorrhea	suicide attempt	414	2926	23	90	--
				disordered eating (restrictive tendency)			104	435	--
				disordered eating (bulimic tendency)			61	596	--
27	Cinar et al.,[43] 2011	311	PCOS	depression	226	85	64	4	n<5
28	Pastore et al.,[44] 2011	190	PCOS	depression symptoms (mild)	94	96	38	28	[Summated into depression total]
				depression symptoms (moderate)			6	18	
				depression symptoms (severe or very severe)			6	3	
				depression total			50	49	--
29	Khandker et al.,[38] 2007	480	vulvodynia	antecedent history of mood disorder (MDD or dysthymia) only	240	240	40	15	--
				antecedent history of anxiety disorders only			12	5	--
				antecedent history of mood and anxiety disorders			12	7	Outcome is subset of antecedent history of mood/anxiety disorders only
				recurrent or new onset of mood disorders (MDD or dysthymia) as a consequence of vulvodynia			35	26	Outcome triggered by explicit event
				recurrent or new onset of anxiety disorders as a			10	6	Outcome triggered by explicit event

© 2023 Zaks N et al. *JAMA Network Open.*

				consequence of vulvodynia					
				recurrent or new onset of mood and anxiety disorders as a consequence of vulvodynia			10	2	n<5
30	Jedel et al.,[45] 2010	60	PCOS	depression	30	30	16	6	--
				anxiety			19	4	n<5
31	Watts et al.,[37] 2010	345	vaginismus	anxiety disorders	244	101	60	17	--
32	Laggari et al.,[46] 2009	44	PCOS	depression (mild)	22	22	6	2	n<5
				depression (moderate)			0	0	n<5
		27	Mayer-Rokitansky-Küster-Hauser Syndrome	depression (mild)	5	22	1	2	n<5
				depression (moderate)			1	0	n<5
33	Romão et al.,[66] 2009	106	CPP	depression	52	54	21	16	--
				anxiety			38	20	--
34	Adali et al.,[47] 2008	84	PCOS	depression	42	49	14	5	--
35	Benson et al.,[48] 2008	85	PCOS	depression	57	28	26	4	n<5
36	Månsson et al.,[49] 2008	98	PCOS	any major depressive episode	49	49	33	17	--
				recurrent depressive episodes			22	9	Outcome is subset of any major depressive episode
				suicide attempt			7	1	n<5
				any manic or hypomanic episode			2	2	n<5
				panic disorder			5	4	n<5
				social phobia			13	1	n<5
				GAD			6	1	n<5
				OCD			1	0	n<5
				any eating disorder			10	2	n<5
				bulimia nervosa			6	2	n<5
37	Hollinrake et al.,[50] 2007	206	PCOS	depressive disorders (unspecified)	103	103	22	3	n<5
				MDD			14	2	n<5
				anxiety disorders			15	1	n<5
				binge eating disorder			13	2	n<5
38	Coleman et al., 2006*	505	genital prolapse	depression	251	254	49	50	Primary diagnosis derives from distinct environmental etiologies

© 2023 Zaks N et al. *JAMA Network Open.*

		496	reproductive tract infection	depression	247	249	48	51	Primary diagnosis derives from distinct environmental etiologies
		506	reproductive-organ masses	depression	93	413	31	68	Primary diagnosis derives from distinct environmental etiologies
		486	cervical dysplasia	depression	29	457	6	91	Primary diagnosis derives from distinct environmental etiologies
		477	pelvic tenderness	depression	51	426	15	80	Primary diagnosis derives from distinct environmental etiologies
39	Himelein et al.,[51] 2006	140	PCOS	depression (moderate to severe)	40	100	11	5	--
40	Lorençatto et al.,[67] 2006	100	endometriosis and CPP	depression (mild)	50	50	17	12	[Summated into depression total]
				depression (moderate/severe)			26	7	
				depression total			43	19	--
41	Jahanfar et al., 2005*	154	subclinical eating disorder	amenorrhea	12	142	2	2	n<5
				anovulation			4	8	n<5
				irregular menstruation			6	40	Outcome derives from distinct environmental etiologies
42	Aikens et al.,[28] 2003	64	vulvar dysesthesia	depression history	32	32	12	11	--
				untreated depressive disorder			9	9	Outcome is subset of depression history
43	Nylanderlundqvist et al., 2003*	60	vulvar vestibulitis	depression	30	30	18	3	n<5
44	Walker et al.,[29] 1995	100	CPP	depression (lifetime)	50	50	32	11	--
				depression (current)			12	2	n<5
				dysthymic disorder			13	1	n<5
				panic disorder (current)			5	1	n<5
				phobia (lifetime)			18	5	--
				somatization (full DSM III)			6	1	n<5
				abridged somatization			32	6	--
45	Hodgkiss et al.,[68] 1994	62	CPP	depression	29	33	11	4	n<5
				anxiety			13	11	--
46	Giles et al., 1993*	17	functional hypothalamic amenorrhea	major depression	9	8	1	1	n<5
				minor depression			1	0	n<5
				labile personality disorder			1	0	n<5
				GAD			1	0	n<5

© 2023 Zaks N et al. *JAMA Network Open.*

		14	organic amenorrhea	dysthymic disorder	6	8	3	0	n<5
				adjustment disorder			1	0	n<5
				phobic disorder			1	0	n<5
47	Slocumb et al.,[69] 1989	82	pelvic pain syndrome	anxiety	41	41	15	4	n<5
				depression			13	2	n<5
48	Harrop-Griffiths et al.,[70] 1988	55	undergone laparoscopy for chronic pelvic pain	depression (lifetime)	25	30	16	5	Nested sample of Walker et al., 1995
				depression (current)			7	1	
				somatization (current)			1	0	
				phobias (current)			8	3	
				panic disorder (current)			2	0	
49	Flint et al., 1983*	24	amenorrhea	schizophrenia	6	18	0	3	n<5
				depression			3	7	n<5
50	Fava et al., 1981*	30	amenorrhea (w/ and w/o hyperprolactinemia)	MDD	20	10	3	0	n<5

** =not included in study/not on reference list; MDD=major depressive disorder; ADHD=attention-deficit/hyperactivity disorder; GAD=generalized anxiety disorder; OCD=obsessive compulsive disorder; ASC=autism spectrum condition; PTSD=post-traumatic stress disorder; NOS=not otherwise specified; TMAS=Taylor Manifest Anxiety Scale; STAI=Spielberger State-Trait Anxiety Inventory.*

© 2023 Zaks N et al. *JAMA Network Open.*

eTable 4. Newcastle-Ottawa Quality Assessment for Case-Control Studies

#	Study	Total N	Primary diagnosis	Outcome diagnosis	N cases	N controls	Included in quantitative synthesis?	Selection	Comparability	Exposure	NOS score
1	Chen, 2020	35872	PCOS	bipolar disorder	7175	28697	Yes	2	2	3	**7**
2	Alur-Gupta, 2019	414	PCOS	depression	189	225	Yes	1	2	2	**5**
				anxiety							
3	Çoban, 2019	59	PCOS	MDD	28	31	No	1	1	1	**3**
				ADHD							
				social anxiety disorder							
				separation anxiety disorder							
				GAD							
				panic disorder							
				specific phobia							
				OCD							
				anorexia nervosa							
				bulimia nervosa							
4	Harnod, 2019	38420	PCOS	anxiety	7684	30736	Yes	2	2	3	**7**
5	Mazi, 2019	200	pelvic floor dysfunction	depression	100	100	No	1	0	1	**2**
6	Meng, 2019	360	postpartum depression	dysmenorrhea	120	240	No	1	2	2	**5**
				low birth weight of child							
				preterm birth of child							
7	Siqueira-Campos, 2019	200	CPP	depression	100	100	Yes	1	2	2	**5**
				anxiety							
				both depression and anxiety							
8	Cherskov, 2018	140918	PCOS phenotype	ASC	26263	130717	Yes	2	2	2	**6**
				depression							
				anxiety							
				schizophrenia							
		5826		PCOS (Read code)	971	4855					

© 2023 Zaks N et al. *JAMA Network Open.*

			Autism Spectrum Condition (ASC)	PCOS (NIH criteria)							
				PCOS (Rotterdam criteria)							
				anovulation							
				hyperandrogenemia							
				polycystic ovaries							
9	Cesta, 2017	12628	PCOS	MDD (lifetime)	752	11876	No	2	2	3	**7**
10	Enjezab, 2017	123	PCOS	depression (mild)	62	61	Yes	1	1	2	**4**
				depression (moderate)							
				depression (severe)							
				depression total							
11	Tan, 2017	220	PCOS	depression (clinical)	120	100	Yes	2	2	2	**6**
				anxiety (trait)							
				anxiety (state)							
				depression symptoms							
12	Akdağ Cirik, 2016	150	PCOS (NIH phenotype)	depression	54	49	Yes	1	2	2	**5**
				anxiety							
			PCOS (non-NIH phenotype)	depression	47	49					
				anxiety							
			PCOS total	depression	101	49					
				anxiety							
13	Cesta, 2016	268235	PCOS	schizophrenia spectrum disorder	24385	243850	Yes	2	2	3	**7**
				schizophrenia, pure							
				bipolar disorder							
				depressive disorders, any							
				severe depression							
				anxiety disorders, any							
				social phobia							
				OCD							
				eating disorders, any							

© 2023 Zaks N et al. *JAMA Network Open.*

				anorexia							
				bulimia							
				personality disorders, any							
				gender identity disorders							
				autism spectrum disorders							
				autism							
				asperger’s syndrome							
				ADHD							
				suicide, attempted							
				suicide, completed							
				alcoholism							
14	De Graaff, 2016	123	endometriosis	anxiety (mild)	83	40	Yes	1	0	1	**2**
				anxiety (moderate)							
				anxiety (severe)							
				anxiety total							
				depression (mild)							
				depression (moderate)							
				depression (severe)							
				depression total							
15	Kayhan, 2016	190	abnormal uterine bleeding	any mood disorder	96	94	Yes	1	2	1	**4**
				major depression							
				dysthymia							
				depression total							
				bipolar disorder							
				any anxiety disorder							
				GAD							
				panic disorder							
				OCD							
				specific phobia							
				social anxiety disorder							

© 2023 Zaks N et al. *JAMA Network Open.*

				anxiety disorder NOS							
				PTSD							
				comorbidity of mood and anxiety disorder							
16	Osório, 2016	100	CPP	current major depressive	50	50	Yes	1	2	2	**5**
				bipolar disorder							
				dysthymia							
				substance abuse/dependence							
				panic							
				obsessive-compulsive							
				post-traumatic stress							
				social anxiety							
				specific phobies							
				any anxiety disorder							
				somatization							
				hypochondria							
				anorexia							
				bulimia							
				eating disorders total							
17	Shen, 2016	43860	pelvic inflammatory disease (PID)	depressive disorder	21930	21930	No	2	2	3	**7**
				anxiety disorder							
				bipolar disorder							
				schizophrenia							
18	Asik, 2015	121	PCOS	depression	71	50	Yes	1	1	2	**4**
				anxiety							
19	Hergüner, 2015	80	PCOS	ADHD (childhood)	40	40	No	1	0	2	**3**
20	Hussain, 2015	150	PCOS	MDD	110	40	No	1	1	2	**4**
				GAD							
				OCD							
				panic disorder							
				suicidality							

© 2023 Zaks N et al. *JAMA Network Open.*

				bipolar affective disorder							
				dysthymia							
				agoraphobia							
21	Iglesias-Rios, 2015	1186	vulvodynia	depression	221	965	Yes	2	2	2	**6**
				PTSD							
		1574	short-term or past vulvar symptoms	depression	609	965					
				PTSD							
22	Davari-Tanha, 2014	220	PCOS	bipolar disorder	110	110	Yes	1	1	2	**4**
				depression							
23	Hung, 2014	27155	PCOS	schizophrenia	5431	21724	Yes	2	2	3	**7**
				bipolar disorder							
				depressive disorder							
				anxiety disorder							
24	Reynolds-May, 2014	139	bipolar disorder	history of oligomenorrhea	103	36	Yes	3	0	1	**4**
				history of amenorrhea							
				current oligomenorrhea							
				current amenorrhea							
				PCOS							
				central amenorrhea							
				menstrual cycle < 25 days							
				menstrual bleeding >10 days							
25	Gagua, 2013	424	primary dysmenorrhea	depression (moderate)	276	148	Yes	1	0	2	**3**
				depression (severe)							
				depression total							
				anxiety (moderate, TMAS)							
				anxiety (high, TMAS)							
				anxiety total							
				anxiety (moderate, STAI)							

© 2023 Zaks N et al. *JAMA Network Open.*

				anxiety (high, STAI)							
26	Ambresin, 2012	3340	severe dysmenorrhea	suicide attempt	414	2926	Yes	2	2	2	**6**
				disordered eating (restrictive tendency)							
				disordered eating (bulimic tendency)							
27	Cinar, 2011	311	PCOS	depression	226	85	No	2	1	2	**5**
28	Pastore, 2011	190	PCOS	depression symptoms (mild)	94	96	Yes	0	2	2	**4**
				depression symptoms (moderate)							
				depression symptoms (severe or very severe)							
				depression total							
29	Khandker, 2007	480	vulvodynia	antecedent history of mood disorder (MDD or dysthymia) only	240	240	Yes	2	2	2	**6**
				antecedent history of anxiety disorders only							
				antecedent history of mood and anxiety disorders							
				recurrent or new onset of mood disorders (MDD or dysthymia) as a consequence of vulvodynia							
				recurrent or new onset of anxiety disorders as a consequence of vulvodynia							
				recurrent or new onset of mood and anxiety disorders as a consequence of vulvodynia							
30	Jedel, 2010	60	PCOS	depression	30	30	Yes	1	2	2	**5**
				anxiety							
31	Watts, 2010	345	vaginismus	anxiety disorders	244	101	Yes	0	0	1	**1**
32	Laggari, 2009	44	PCOS	depression (mild)	22	22	No	2	2	1	**5**

© 2023 Zaks N et al. *JAMA Network Open.*

				depression (moderate)							
		27	Mayer-Rokitansky-Küster-Hauser Syndrome	depression (mild)	5	22					
				depression (moderate)							
33	Romão, 2009	106	CPP	depression	52	54	Yes	1	0	2	**3**
				anxiety							
34	Adali, 2008	84	PCOS	depression	42	49	Yes	1	1	2	**4**
35	Benson, 2008	85	PCOS	depression	57	28	No	1	1	2	**4**
36	Månsson, 2008	98	PCOS	any major depressive episode	49	49	Yes	2	1	2	**5**
				recurrent depressive episodes							
				suicide attempt							
				any manic or hypomanic episode							
				panic disorder							
				social phobia							
				GAD							
				OCD							
				any eating disorder							
				bulimia nervosa							
37	Hollinrake, 2007	206	PCOS	depressive disorders (unspecified)	103	103	No	1	1	2	**4**
				MDD							
				anxiety disorders							
				binge eating disorder							
38	Coleman, 2006	505	genital prolapse	depression	251	254	No	2	2	2	**6**
		496	reproductive tract infection	depression	247	249					
		506	reproductive-organ masses	depression	93	413					
		486	cervical dysplasia	depression	29	457					
		477	pelvic tenderness	depression	51	426					

© 2023 Zaks N et al. *JAMA Network Open.*

39	Himelein, 2006	140	PCOS	depression (moderate to severe)	40	100	Yes	2	2	2	**6**
40	Lorençatto, 2006	100	endometriosis and CPP	depression (mild)	50	50	Yes	1	0	2	**3**
				depression (moderate/severe)							
				depression total							
41	Jahanfar, 2005	154	subclinical eating disorder	amenorrhea	12	142	No	1	2	2	**5**
				anovulation							
				irregular menstruation							
42	Aikens, 2003	64	vulvar dysesthesia	depression history	32	32	Yes	1	2	2	**5**
				untreated depressive disorder							
43	Nylanderlundqvist, 2003	60	vulvar vestibulitis	depression	30	30	No	1	1	2	**4**
44	Walker, 1995	100	CPP	depression (lifetime)	50	50	Yes	2	2	2	**6**
				depression (current)							
				dysthymic disorder							
				panic disorder (current)							
				phobia (lifetime)							
				somatization (full DSM III)							
				abridged somatization							
45	Hodgkiss, 1994	62	CPP	depression	29	33	Yes	1	0	2	**3**
				anxiety							
46	Giles, 1993	17	functional hypothalamic amenorrhea	major depression	9	8	No	1	2	2	**5**
				minor depression							
				labile personality disorder							
				GAD							
		14	organic amenorrhea	dysthymic disorder	6	8					
				adjustment disorder							
				phobic disorder							
47	Slocumb, 1989	82	pelvic pain syndrome	anxiety	41	41	No	1	2	2	**5**
				depression							

© 2023 Zaks N et al. *JAMA Network Open.*

48	Harrop-Griffiths, 1988	55	undergone laparoscopy for chronic pelvic pain	depression (lifetime)	25	30	No	1	0	2	**3**
				depression (current)							
				somatization (current)							
				phobias (current)							
				panic disorder (current)							
49	Flint, 1983	24	amenorrhea	schizophrenia	6	18	No	1	0	2	**3**
				depression							
50	Fava, 1981	30	amenorrhea (w/ and w/o hyperprolactinemia)	MDD	20	10	No	1	2	2	**5**

© 2023 Zaks N et al. *JAMA Network Open.*

eFigure 1. Funnel Plot of Included Studies on Psychiatric Outcome Based on Reproductive System Disorder Status

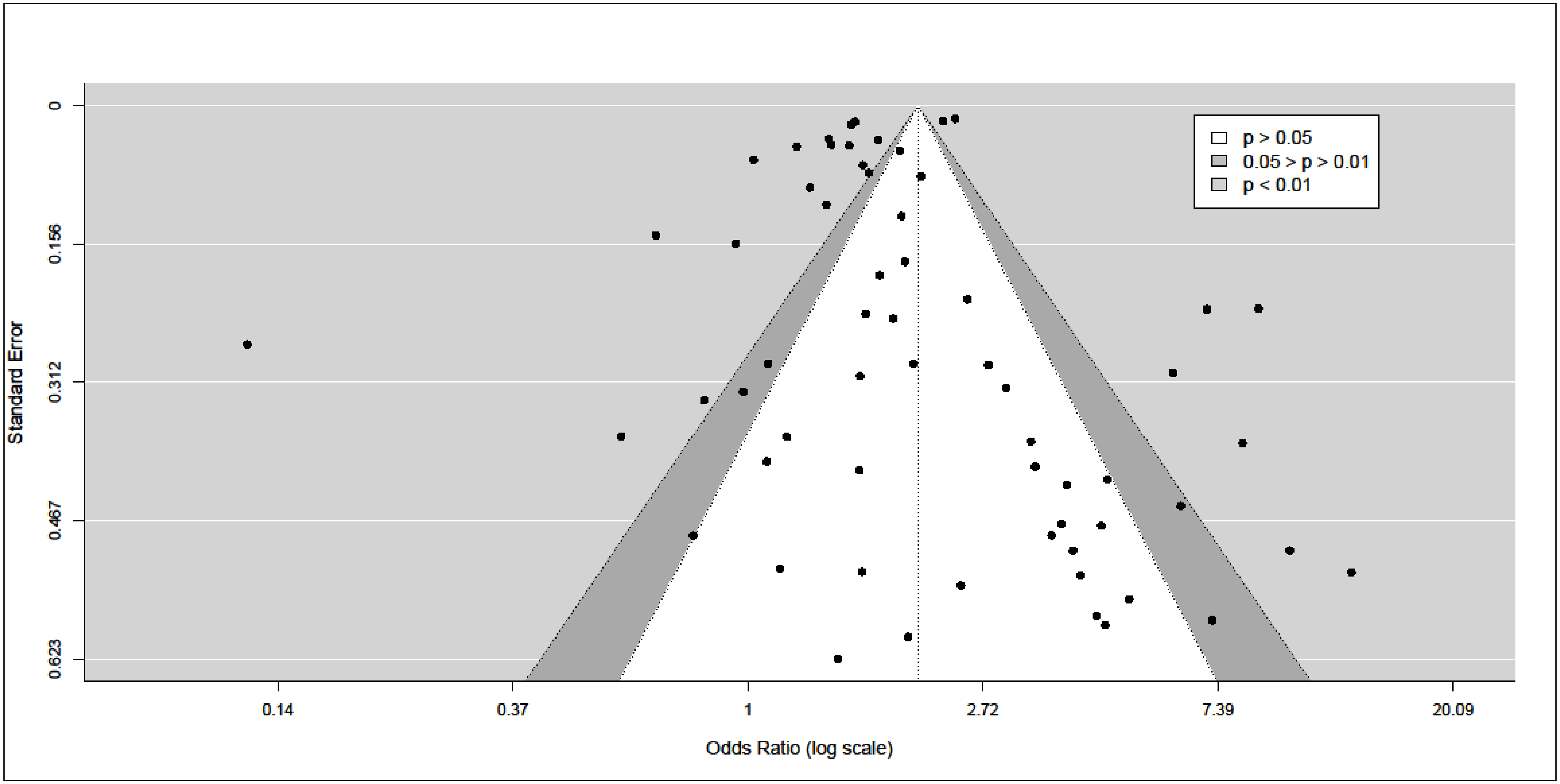

© 2023 Zaks N et al. *JAMA Network Open.*
