# Supplementary material for: Association Between Mental Health and Reproductive System Disorders in Women: A Systematic Review and Meta-analysis
Source: JAMA Netw Open. 2023 Apr 18;6(4):e238685. doi: 10.1001/jamanetworkopen.2023.8685 (PMC10114079; doi:10.1001/jamanetworkopen.2023.8685)
Supplement: Supplement 2. — Data Sharing Statement [file jamanetwopen-e238685-s002.pdf]

Data Sharing Statement

Zaks. Association Between Mental Health and Reproductive System Disorders in Women: Systematic Review and Meta-analysis. *JAMA Netw Open*. Published April 18, 2023. doi:10.1001/jamanetworkopen.2023.8685

Data
Data available: No
